# Structural Features of the Regulatory ACT Domain of Phenylalanine Hydroxylase

**DOI:** 10.1371/journal.pone.0079482

**Published:** 2013-11-14

**Authors:** Carla Carluccio, Franca Fraternali, Francesco Salvatore, Arianna Fornili, Adriana Zagari

**Affiliations:** 1 CEINGE-Biotecnologie Avanzate, S.c.a r.l., Napoli, Italy; 2 Dipartimento di Medicina Molecolare e Biotecnologie Mediche, Università degli studi di Napoli “Federico II”, Napoli, Italy; 3 Randall Division of Cell and Molecular Biophysics, King’s College London, London, United Kingdom; 4 **SDN**-Istituto di Ricerca Diagnostica e Nucleare, Napoli, Italy; University of Canterbury, New Zealand

## Abstract

Phenylalanine hydroxylase (PAH) catalyzes the conversion of L-Phe to L-Tyr. Defects in PAH activity, caused by mutations in the human gene, result in the autosomal recessively inherited disease hyperphenylalaninemia. PAH activity is regulated by multiple factors, including phosphorylation and ligand binding. In particular, PAH displays positive cooperativity for L-Phe, which is proposed to bind the enzyme on an allosteric site in the N-terminal regulatory domain (RD), also classified as an ACT domain. This domain is found in several proteins and is able to bind amino acids. We used molecular dynamics simulations to obtain dynamical and structural insights into the isolated RD of PAH. Here we show that the principal motions involve conformational changes leading from an initial open to a final closed domain structure. The global intrinsic motions of the RD are correlated with exposure to solvent of a hydrophobic surface, which corresponds to the ligand binding-site of the ACT domain. Our results strongly suggest a relationship between the Phe-binding function and the overall dynamic behaviour of the enzyme. This relationship may be affected by structure-disturbing mutations. To elucidate the functional implications of the mutations, we investigated the structural effects on the dynamics of the human RD PAH induced by six missense hyperphenylalaninemia-causing mutations, namely p.G46S, p.F39C, p.F39L, p.I65S, p.I65T and p.I65V. These studies showed that the alterations in RD hydrophobic interactions induced by missense mutations could affect the functionality of the whole enzyme.

## Introduction

Phenylalanine hydroxylase (PAH) is an iron-containing enzyme, mainly expressed in liver, that catalyzes the conversion of the essential amino acid L-Phe (hereafter referred to as “Phe”) into L-Tyr utilizing the cofactor 6R-L-erythro-tetrahydrobiopterin (BH4) and dioxygen [1], [2]. Defects in PAH enzymatic activity caused by mutations in the human gene result in an autosomal recessively inherited disorder of amino acid metabolism known as hyperphenylalaninemia (HPA). Impaired PAH function results in the accumulation of high levels of blood plasma phenylalanine and of its neurotoxic metabolites [3].

Mutations in the human *PAH* gene lead to a variety of clinical and biochemical phenotypes that differ in severity [4], [5]: mild hyperphenylalaninemia, mild phenylketonuria and classical phenylketonuria (PKU). Early diagnosis and prompt intervention has allowed most individuals with PKU to avoid severe mental disability [6]. Many countries now include a test for HPA in neonatal screening programs. To prevent mental retardation due to the buildup of neurotoxic metabolites of Phe, patients with severe PKU must be treated with a low-Phe diet starting early in life [7]. Interestingly, Phe excess is a main cause of such alterations in brain function as spatial learning deficits and long-term potentiation [8], thus indirectly indicating that a single amino acid, such as Phe, can alter physiological homeostasis probably by unfavorably interacting with the functionality of cell proteins.

Generally, *PAH* mutations result in reduced enzyme activity [9]–[11] and stability [12], [13], and some alter its oligomeric state [13], [14]. It is now generally recognized that there is relationship between structural alteration and phenotype. To date, more than 560 mutations in the *PAH* gene have been identified (see PAH Mutation Analysis Consortium database: http://www.pahdb.mcgill.ca/) and they are spread throughout the 3D structure, although most of them are located in the catalytic domain.

**Figure 1:**
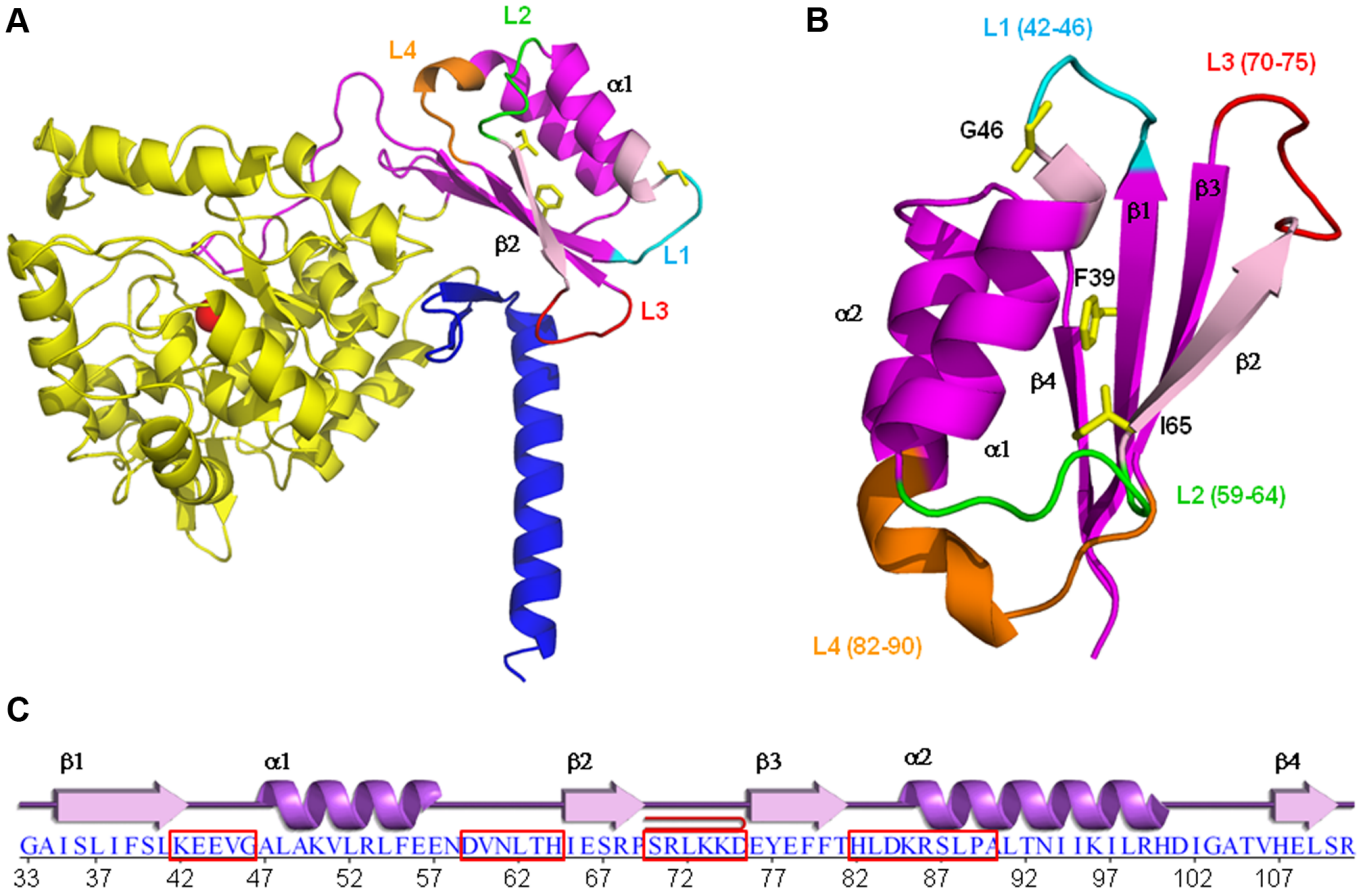
Structure representation of the human PAH subunit and its RD. A) Structural model of a full-length PAH subunit created by superimposing the catalytic domains of the dimeric rat crystal structure (1PHZ), which includes the RD (19–117), and of the tetrameric human crystal structure (2PAH), which includes the catalytic/tetramerization domain. RD is drawn in magenta, the catalytic domain in yellow and the tetramerization domain in blue. The iron is shown as a red sphere. B) Starting models of the ACT domain (33–111 residues) of wt-hPAH with the residues G46, F39 and I65 drawn as a yellow stick. In light magenta are the two highly conserved GAL and IERSP motifs. The mobile loop-containing regions L1 to L4 are drawn in different colors. C) Primary and secondary structure of the human RD represented in B. Loop-containing regions L1 to L4 are boxed in red.

Phenylalanine hydroxylase is a tetrameric enzyme assembled as a dimer of dimers. Each monomer (Figure 1A) consists of 452 amino acids (about 52 kDa), adopts an α/β structure and is built up from an N-terminal regulatory domain (RD) (residues 1–117 in the human enzyme), a catalytic domain (residues 118–410), which includes binding sites for iron, substrate and cofactor, and a tetramerization domain (residues 411–452). Crystal structure information is available for several truncated forms of human PAH (hPAH), with or without cofactor and substrate analogs, but the full-length structure has not yet been solved, neither has the structure of the RD of hPAH. The RD from rat dimeric PAH is the closest homolog of the hPAH RD for which structural data are available (PDB code 1PHZ) [15], and it shares a 84% sequence identity with the RD of hPAH. In the known rPAH crystal structure, however, the N-terminal fragment (residues 1–32) is unstructured.

The N-terminal RD of PAH contains an ACT domain (residues 33–111), a structural motif composed of four β-strands and two α-helices arranged in a βαββαβ fold (Figures 1B, C). The ACT domain was identified by sequence analysis of a set of proteins involved in amino acid and purine metabolism and regulated by specific amino acids [16]. In the available structures, this domain was found either isolated or associated into oligomers of 2, 3 or 4 units. Differently, in the tetrameric PAH structure, the four ACT domains do not associate with each other. In many cases, the proteins containing the ACT domain are allosteric enzymes regulated *via* transmission of conformational changes upon ligand binding [16]. In the ACT-domain-containing proteins, the ligands usually bind at the interfaces between ACT domains and in proximity of a specific Gly (Gly46 in hPAH) located in the loop between the first β-strand and the first α helix [17].

The regulatory function of the N-terminal ACT domain of PAH is poorly understood. The major regulatory mechanisms of PAH include activation by phenylalanine, inhibition by BH4, and additional activation by phosphorylation [2]. Tetrameric wild-type PAH displayed substrate activation and positive cooperativity for Phe binding involving all three functional domains and all four subunits in the holoenzyme [1]. BH4 acts as a negative regulator by blocking Phe activation [1], [18]. Indeed, the binding of BH4 to PAH without Phe leads to the formation of an inactive PAH-BH4 complex. However, recent studies demonstrate that the binding of BH4 to a Phe-activated form of PAH results in positive cooperativity [19]. Phosphorylation acts as a mediator of Phe activation by decreasing the Phe concentration required to activate the enzyme [1], [18].

Various, conflicting, models have been proposed to explain these mechanisms. One possible explanation is that the activation mechanism results from homotropic binding of Phe at the active site and the RD is involved in cooperativity through its interactions with the catalytic and oligomerization domains [20]–[23]. Other groups proposed that Phe binds to an allosteric site, besides to the active site, on the RD of PAH, thereby inducing large conformational changes [1], [18], [24]. Support for the latter model comes from the finding that when the RD is removed, hPAH is no longer activated by preincubation with the substrate [12]. Recently, nuclear magnetic resonance spectroscopy provided strong evidence for a Phe-binding site in the isolated RD [25]. Moreover, the GAL (46–48) and IESRP (65–68) sequence motifs, conserved in the ACT domain family, are involved in Phe binding at the PAH RD [26]. A Phe-bound structure of the protein prephenate dehydratase (PDT) from *Chlorobium tepidum TLS* is available (PDB code 2QMX) in which the ligand-binding region in the ACT domain contains the GAL-IESRP motif [27]. While Phe binds PDT at the dimer interface of two ACT domains, the GAL-IESRP motif in PAH is at the interface between the RD and the catalytic domain of the adjacent subunit. The structural similarities between the above-mentioned allosteric enzymes and the ACT regulatory domains prompted the hypothesis that Phe binds PAH between the RD and the interacting catalytic domain, near the sequence binding motif [2], [15].

Notwithstanding this large body of data, the putative allosteric Phe-binding site in PAH remains unknown, and the role of the domain as an allosteric module is unclear. In this study, we analyzed, for the first time, the intrinsic structural and dynamical properties of the isolated RD of hPAH using molecular dynamics (MD) simulations in solution, also to determine whether or not it contains an allosteric Phe-binding site. We also investigated the structural and dynamic effects induced by six selected missense disease-causing mutations found in young PKU patients (p.G46S, p.F39C, p.F39L, p.I65S, p.I65T and p.I65V) [28] (see PAH Mutation Analysis Consortium database: http://www.pahdb.mcgill.ca/) and located on the RD of the hPAH enzyme.

## Materials and Methods

### Molecular Modeling

Models of the ACT domain (residues 33–111) of the wt-hPAH enzyme and hPAH mutants were generated *in silico* with the Modeller 9v8 program [29] using the rat wt-rPAH crystal structure as template (PDB code 1phz) [15]. The RD domains of the wt-hPAH and wt-rPAH enzymes share a sequence identity of 84%. The template and the target sequences were aligned using Bodil [30]. Subsequently, the alignment files were used as input in Modeller9v8, and 200 models of each of the 7 PAH forms studied (wt-hPAH and 6 hPAH mutants) were generated. In the case of mutants, the mutated residues were replaced in the alignment file. The program selects the most abundant conformers of the replaced residue and performs a simulated annealing procedure to optimize side chain conformations. The model that presented the best Modeller DOPE score was selected for MD simulations.

The same procedure was used to generate the model of the rat PAH enzyme used to test the stability of an alternate conformation of the D59-H64 (L2) region in wt-rPAH (Figure 1B). In this case, two templates were used: the rat crystallographic structure of PAH for residues 33–57 and 67–111, and the ACT domain (fragment 339–408) of D-3-phosphoglycerate dehydrogenase from *Escherichia coli* (PDB code 1PSD) for residues 58–66. In addition, a tetrameric model of wt-hPAH was built with Modeller 9v8, using as template the regulatory domain from the rat crystal structure (PDB code 1PHZ) and the catalytic/tetramerization domain from the human crystal structure (PDB code 2PAH). Also in this latter case the template and the target sequences were aligned using Bodil.

### Molecular Dynamics Simulations

Molecular Dynamics simulations were performed with the GROMACS 4.0 [31] package using the GROMOS 43A1 force field [32]. Each of the initial models generated *in silico* and the wt-rPAH structure were solvated in boxes containing simple point charge (SPC) water molecules [33], with the minimal distance between the protein and the box boundaries set at 15 Å. Simulations were carried out with periodic boundary conditions and a 2-fs time step. All the bonds in the protein were frozen with the LINCS method [34], while SETTLE [35] was used for water molecules. The Berendsen algorithm [36] was applied for temperature and pressure coupling. The particle mesh Ewald method [37] was used to calculate the electrostatic contribution to non-bonded interactions with a direct sums cut-off of 14 Å. The same cut-off was used for van der Waals interactions. The systems were energy minimized using the GROMOS 43A1 force field with 1000 steps of the steepest descent method and subsequently with 1000 steps of the conjugate gradient method. After minimization, harmonic positional restraints (with an initial force constant of 4.8 kcal/mol/Å^2^) were imposed on the protein heavy atoms and gradually turned off in 360 ps, while the temperature was increased from 200 to 300 K at a constant volume. The systems were then equilibrated for 2 ns without restraints in NPT conditions (T = 300 K, p = 1 bar). Finally, each NPT MD simulation was performed for 50 ns. Two replicas were run for each system using different sets of initial velocities. The total simulation time summed over all systems and replicas was 800 ns. Molecular dynamics trajectories were analyzed using GROMACS 4.0 analysis tools. Images were produced with the Visual Molecular Dynamics software [38] and PyMOL [39].

### Analysis Tools

The secondary structure content was measured with DSSP [40], while the solvent accessible surface area was calculated with the GROMACS utility g_sas tool [41]. Correlated motions were identified by performing Essential Dynamics (ED) [42] on each MD trajectory. Principal components (PCs) were generated by diagonalizing the covariance matrix of C^α^ positions. Correlation webs, connecting C^α^ atom pairs with a correlation (per atom normalized covariance) higher than 0.3 were generated with Dynatraj [43]. A combined ED analysis [44] was also performed on the pseudo-trajectory obtained by concatenating the 16 trajectories of equivalent C^α^ atoms obtained from all the simulated systems (2 replicas x [wt-hPAH+wt−rPAH +6 hPAH mutants]). The equivalence between hPAH and wt-rPAH C^α^ atoms was derived from the sequence alignment. This frequently used type of analysis [45], [46] enables us to easily compare the portions of the conformational space sampled by different simulations and to capture the directions in this space along which the simulated systems mainly differ.

To investigate further the accessible collective motions of the RD, we sampled alternative ensembles of structures for all the models with the tCONCOORD method [47]. tCONCOORD allows computationally efficient sampling of conformational transitions of a protein based on geometrical constraints as determined from the initial coordinates and interaction types. Under-wrapped hydrogen bonds are detected and modeled as unstable. Each model was first energy-minimized using the OPLS-AA force field [48] with 250 steps of the steepest descent algorithm. The OPLSA-AA force field was used in this case to ensure full consistency with tCONCOORD [47]. Ensembles of 1000 structures were then generated using standard tCONCOORD parameters [47]. The similarity of the MD and tCONCOORD correlated motions from ED confirmed that all the relevant loop collective motions had already been sampled by MD.

To investigate interactions with the solvent during simulations, we generated water density maps g(**r**) around each construct [49]–[51]. The maps were calculated at discrete points **r** defined by a 0.5-Å-spaced rectangular grid around the solute. Each structure of the last 2 ns of the trajectory was superimposed on a reference to remove the overall roto-translational motion of the protein. The number density of the water oxygen atoms was then averaged at each grid point over the MD snapshots extracted every 0.1 ps and normalized by the bulk density evaluated in the 6–8 Å shell around the solute. The hydration sites were then identified as local maxima of the density map with g(**r**) >1 and used to define the atomic hydration score *S_hyd_* ^atom^ as previously described [52]. Atoms with a high S_hyd_^atom^ score are either surrounded by many maxima or close to a few maxima with a high density.

The relationship between the RD collective dynamics and the putative Phe binding site was investigated using functional mode analysis (FMA) [53]. This procedure serves to detect the collective motion that is maximally correlated with a particular protein functional property (Maximally Correlated Motion or MCM). The MCM is usually expressed as a linear combination of Principal Components (PCs) derived from ED. We selected the FMA approach in which the MCM coefficients are determined by maximizing the Pearson correlation coefficient between the time evolution of the chosen functional property (solvent accessibility of the putative Phe binding site) and the projection of the trajectory onto the MCM. For each MD simulation, the MCM was expanded in the essential space of the C^α^ atoms, composed of the first *n* PCs accounting for 90% of the total fluctuation. The first 45 ns were used as model building set, while the remaining 5 ns were used for cross-validation.

## Results

*In silico* models were generated for the ACT regulatory domain (residues 33–111) of the wt-hPAH enzyme and of the six hPAH mutants, G46S, F39C, F39L, I65S, I65T and I65V. The model consists of a common α-β sandwich motif with a four-stranded antiparallel β-sheet flanked on one side by two short α-helices (Figure 1A–C). The loop-containing regions L42-V45 (L1), D59-H64 (L2), S70-D75 (L3) and H82-V90 (L4) are highlighted in Figure 1A and B. Two replicas of MD simulations were performed on the generated models and the wt-rPAH template. During the 50-ns-long simulations, the systems showed no large deviations from the initial structures, and at the end of the simulations all trajectories reach a plateau within 2–3 Å in the RMSD from the initial structure calculated over C^α^ atoms (Table S1).

### Mobility and Conformational Change of Loop-containing Regions

**Figure 2:**
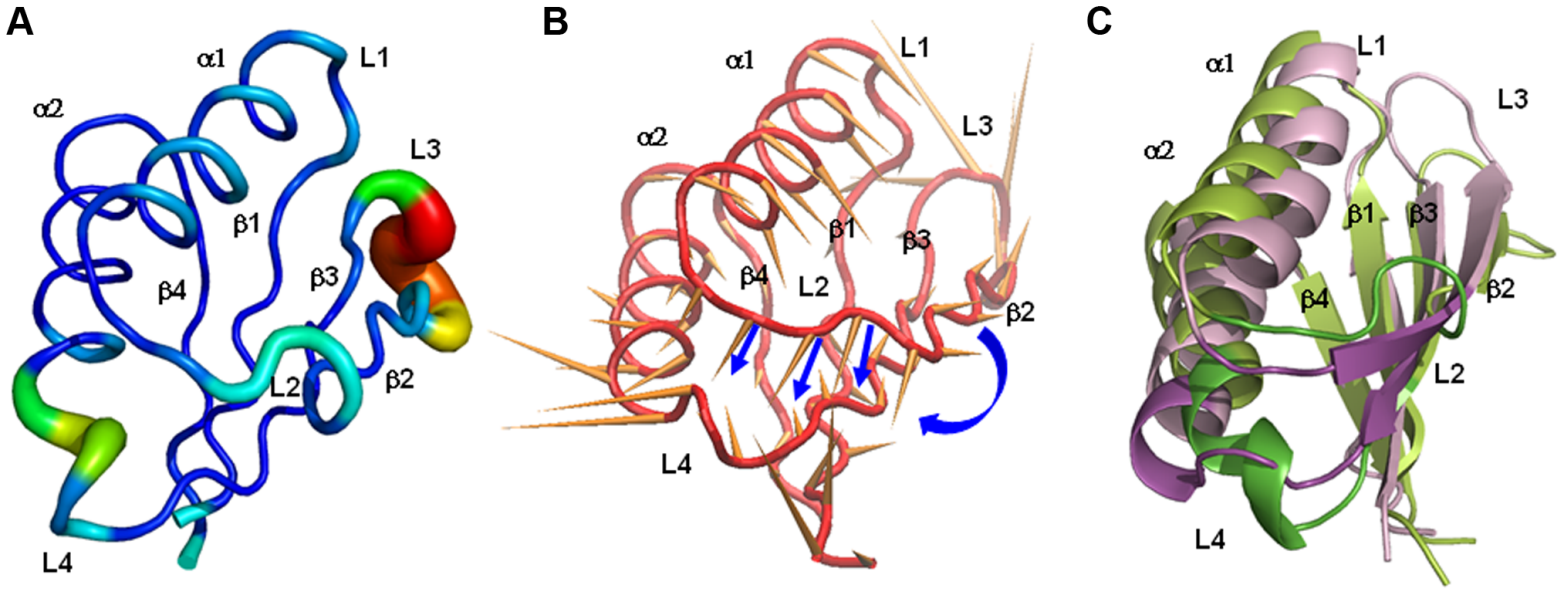
Mobility and conformational change of loop-containing regions. A) Sausage representation of the wt-PAH initial structure, in which the thickness of the sausage is proportional to the root mean square fluctuation of C^α^ atoms from their average positions. Average positions are calculated after least-squares fit superimposition of the trajectory structures onto the starting structure used as reference. B) Porcupine representation of the first PC (accounting for 78% of the overall fluctuation of C^α^ atoms) extracted from the simulation of the wt-hPAH. The orange cones, attached to the average positions of each C^α^ atom, point in the direction of motion described by PC1. Blue arrows highlight the motion corresponding to the transition of L2 to β-strand. C) Initial (light green) and final (light pink) wt-hPAH structures with the L2 and L4 regions drawn in green and magenta.

The dynamics of wt-hPAH is mostly characterized by the large motions of the L2, L3 and L4 regions (Figures 2A, B and S1) and a small fluctuation of L1. In particular, there is a large rearrangement of the L3 loop, located near the dimerization interface in the dimer/tetramer structures. This motion is probably enhanced by the absence of the other subunits in the simulated system. In the rat dimeric PAH crystal structure, the L1 and L3 loops of the RD interact with both the catalytic domain of the opposite subunit and the tetramerization domain of the same subunit. Differently, in the simulated systems, in which the RD is isolated, L1 and L3 are free to move so that we can study their intrinsic flexibility. Regions L2 and L4 are located close to each other on the opposite side of the domain with respect to the L3 loop (Figures 1A, B). The L2 region corresponds to the loop/turn located between helix α1 and the strand β2, while the L4 region includes the β3-α2 connecting loop and the N-terminal part of helix α2 (Figures 1B, C).

**Figure 3:**
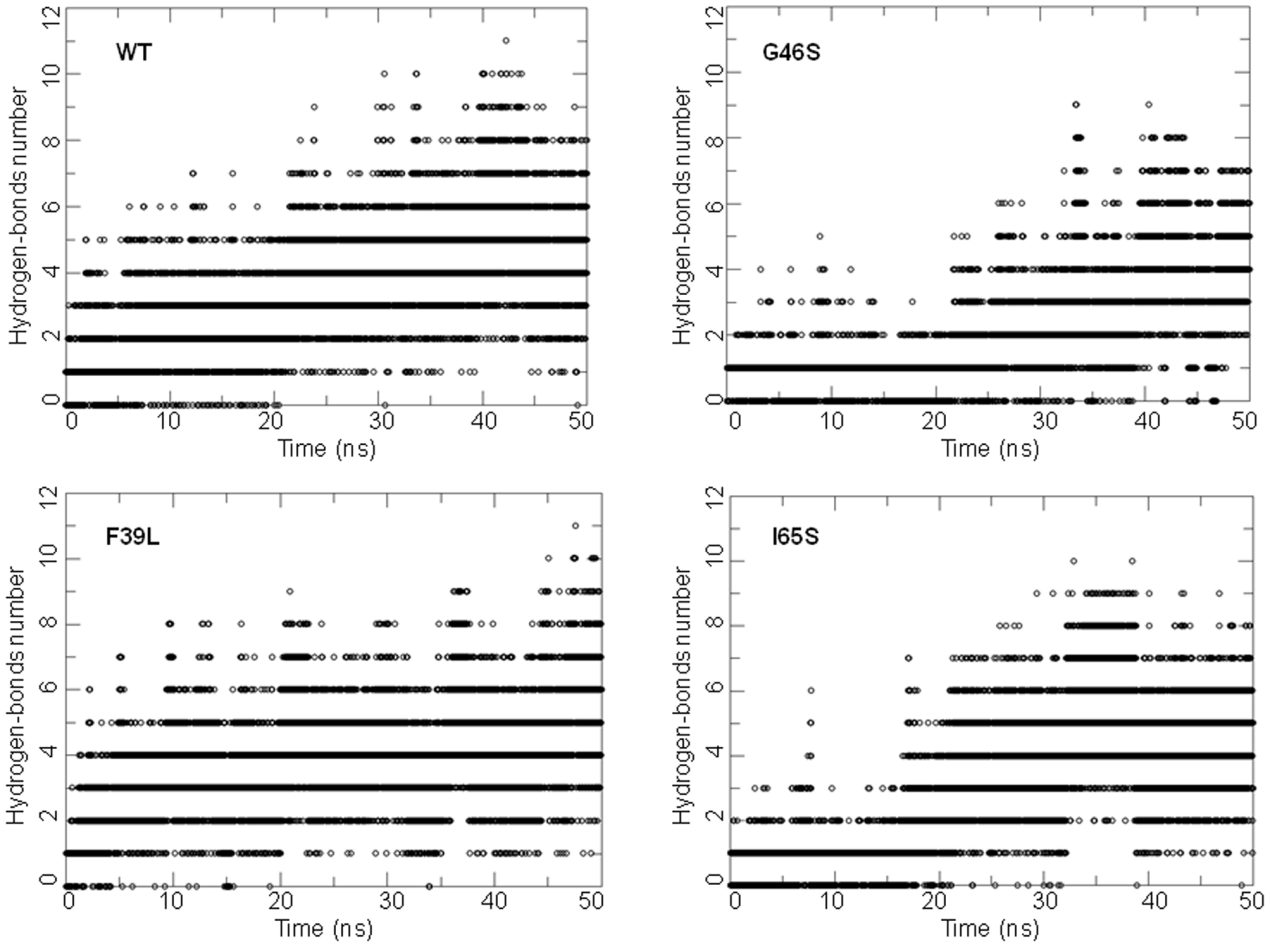
Time-evolution of H-bond number. The panels show the number of H-bonds between the L2 and the L4 region residues (backbone and side chains atoms) *versus* time in the wt-hPAH and in the G46S, F39L, I65S mutants respectively. H-bonds were determined using a cut-off of 30 degrees on the Acceptor-Donor-Hydrogen angle and of 3.5 Å on the Acceptor-Donor distance.

As shown by ED analyses, performed on each system, the movements of regions L2, L3 and L4 are represented by the first PC of the motion in the human wild-type enzyme (Figure 2B). In particular, part of the L2 region (residues from 61 to 64) changes its conformation from turn to β-strand, thus resulting in elongation of the β2 strand, while the L4 region undergoes unwinding of the N-terminal part of helix α2, which brings the two regions closer to each other (Figures 2A–C). This is in line with the formation of new H-bonds between the L2 and L4 regions observed during the dynamics (as shown in the WT panel of Figure 3). Indeed, His64 (L2) and Thr63 (L2) form H-bonds with His82 (L4), while Asn61 (L2) interacts with Asp84 (L4).

The conformational change of the L2 region from turn/bend to β-strand in the wt-hPAH is also in line with the secondary structure analysis by DSSP of the simulated trajectories (WT in Figure S2). This change is paralleled by stabilization of the C-terminal part of the β3 strand (Figure S2) that interacts with the L2 region, thereby forming a hydrogen-bond pattern typical of a β-sheet secondary structure (Figure S3).

Similar features were observed for most of the mutants by RMSF, secondary structure analyses and ED analyses performed on each system (Figures 3, S1, S2). In particular, the conformational change of the L2 region from turn/bend to β-strand was found in all systems except in the rat structure and in the F39C mutant (Figure S2). However, the closeness of L2 and L4 and the formation of new H-bonds between these two regions occur in all systems in at least one replica (see Figure 3 for examples). Moreover, in the rat structure and in mutants G46S, F39L, I65T and I65V, also the L1 loop, located near L3, shows a rearrangement (Figure S1).

**Figure 4:**
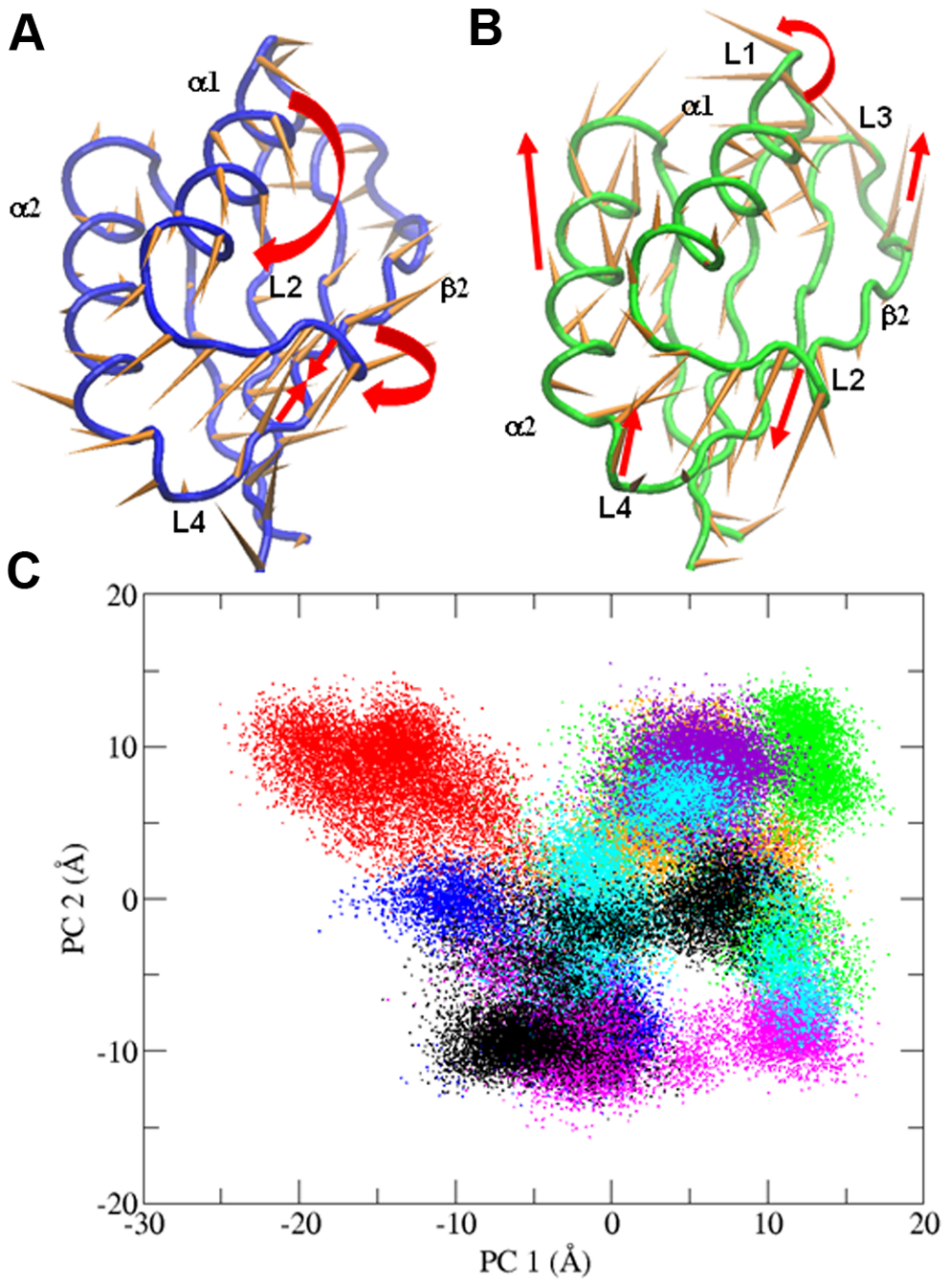
Combined essential dynamics analysis. A) Porcupine plots illustrating the conformational changes represented by the first and B) the second PCs from the combined ED analysis. Each C^α^ atom has a cone pointing in the direction of conformational change described by each PC. C) Projections of each trajectory along PC1 and PC2 from the combined ED. Color code: wt-hPAH in black, wt-rPAH in red, G46S in orange, F39C in blue, F39L in green, I65T in violet, I65S in magenta, I65V in cyan.

To identify inter-simulation similarities and differences among the conformational states of all the studied systems, we performed a “combined essential dynamics” analysis on the concatenated MD trajectories of all enzymes for both replicas (see Materials and Methods). The PCs derived from this analysis define the directions in the conformational space along which the different simulations show the largest variability. Differently from the PCA analysis described above (Figure 2B), these PCs do not represent the essential motions of a specific simulation, but describe the most important conformational variations found across all the different simulations. The first principal component (PC1) describes the transition to β strand of the L2 region (β2 elongation), the nearing of regions L2 and L4 and the reorientation of the α1 helix (Figure 4A). The second principal component (PC2) describes the conformational variability of all loops, and shows a smaller relative contribution of the β strands and α1 versus PC1, and a larger relative contribution of L1 and α2 (Figure 4B). The projection of the MD trajectories onto the essential space (planes) defined by PC1 and PC2 shows that the human and rat wild-type forms explore different regions (Figure 4C). In particular, the human wild-type samples regions in the conformational space where the elongation of β2 occurs (PC1>0), while in the wt-rPAH L2 remains in a turn conformation (PC1<0) during both simulations. The regions at PC1>0 are sampled by all mutants, except F39C, in at least one replica. The largest differences between mutants and human wild-type enzymes are observed along the second PC. Indeed, mutants G46S, F39L, I65T and I65V, in which α2 and all loops (including L1) have a higher conformational variability with respect to the β-sheet (Figure S1), explore large portions of the space with PC2>0, which seems inaccessible to the human wild-type and the F39C and I65S mutants (Figure 4C). Though some mutants show similar features, e.g. G46S, I65T and I65V can be grouped together (see also “Analysis of core interactions” below), each mutant has a distinct dynamic behavior.

### L2 Conformation in the RD Structure from wt-rPAH

In the rat RD crystal structure, the L2 region has a particular β-turn conformation (Asn61-His64 turn type IV, Leu62-Ile65 turn type I) and, unlike the human RD, did not undergo the conformational change of this region from turn to β-strand during the simulations (Figures 4C and S2). Screening of all the available structures of other ACT domains (PDB codes: 1PSD, 1RWU, 1TDJ, 1O8B, 1Q5Y, 1I1G, 1NH7, 1ZPV, 1U8S, 1Y7P, 2F1F, 2F06, 2CDQ, 2QMX) showed that the L2 region preferentially adopts the β-strand conformation, while the turn conformation of the L2 region is specific to the rat RD. The B-factor values of the atoms belonging to the L2 region in the rat PAH crystal structure (PDB code 1PHZ) are higher than the average B-factor value of the protein. This suggests that the L2 region is quite flexible in the crystal.

**Figure 5:**
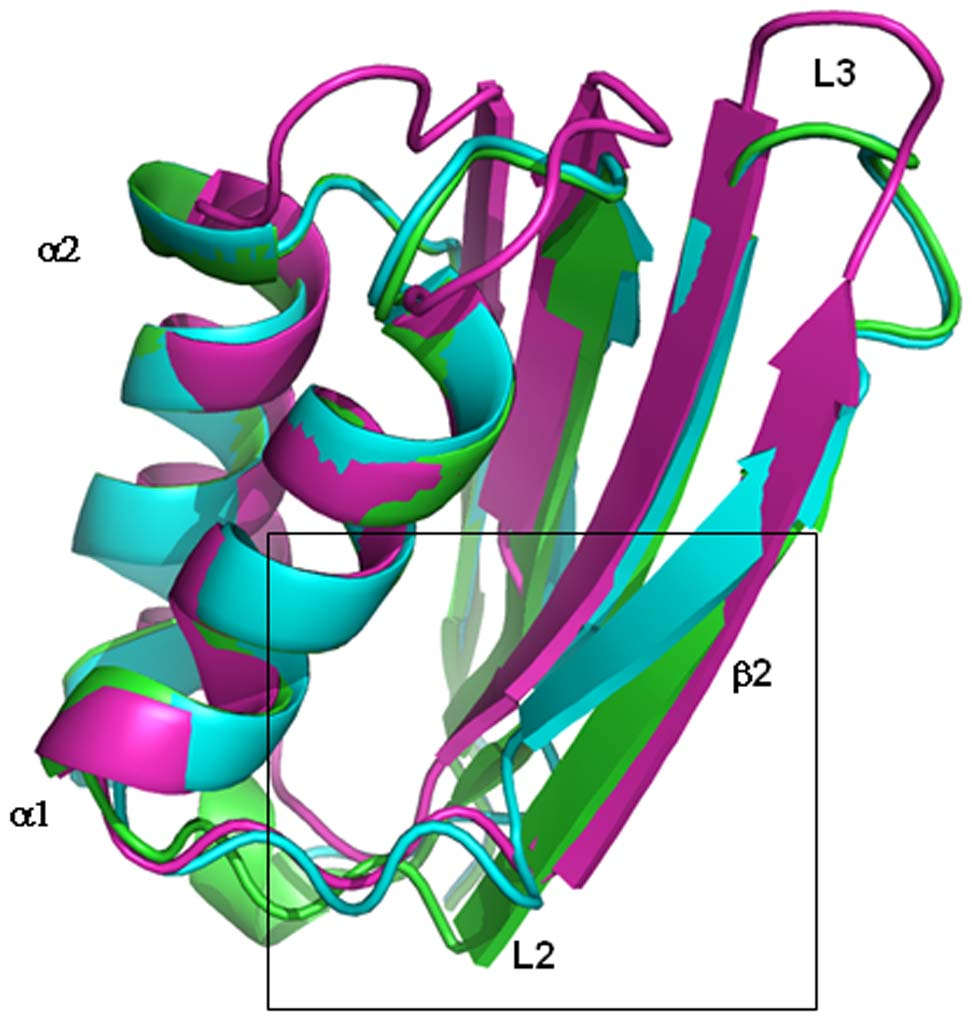
Superimposition of the ACT domains. Superimposition of the ACT domains of D-3-phosphoglycerate dehydrogenase from *Escherichia coli* (magenta), crystallographic wt-rPAH (cyan) and *in silico* model of wt-rPAH (green). The L2 region discussed in the article is boxed.

To test the stability of a hypothetical β conformation of the L2 region in the wt-rPAH, we generated a model of the RD of the rat enzyme using as template the ACT domain of D-3-phosphoglycerate dehydrogenase (3PGDH) from *Escherichia coli*. A superposition of the RD domain from the rat crystal structure, the rat modeled structure and the 3PGDH crystal structure is shown in Figure 5. The overall fold is very similar, apart from the L2 region. Indeed, like the template, the model has the L2 region in a β-strand conformation. We then subjected the model to 50-ns-long MD simulations, and the β-strand conformation of the L2 region was found to be stable throughout the simulation (rat model in Figure S2).

### Correlation between a Hydrophobic Surface and Motion in the RD

**Figure 6:**
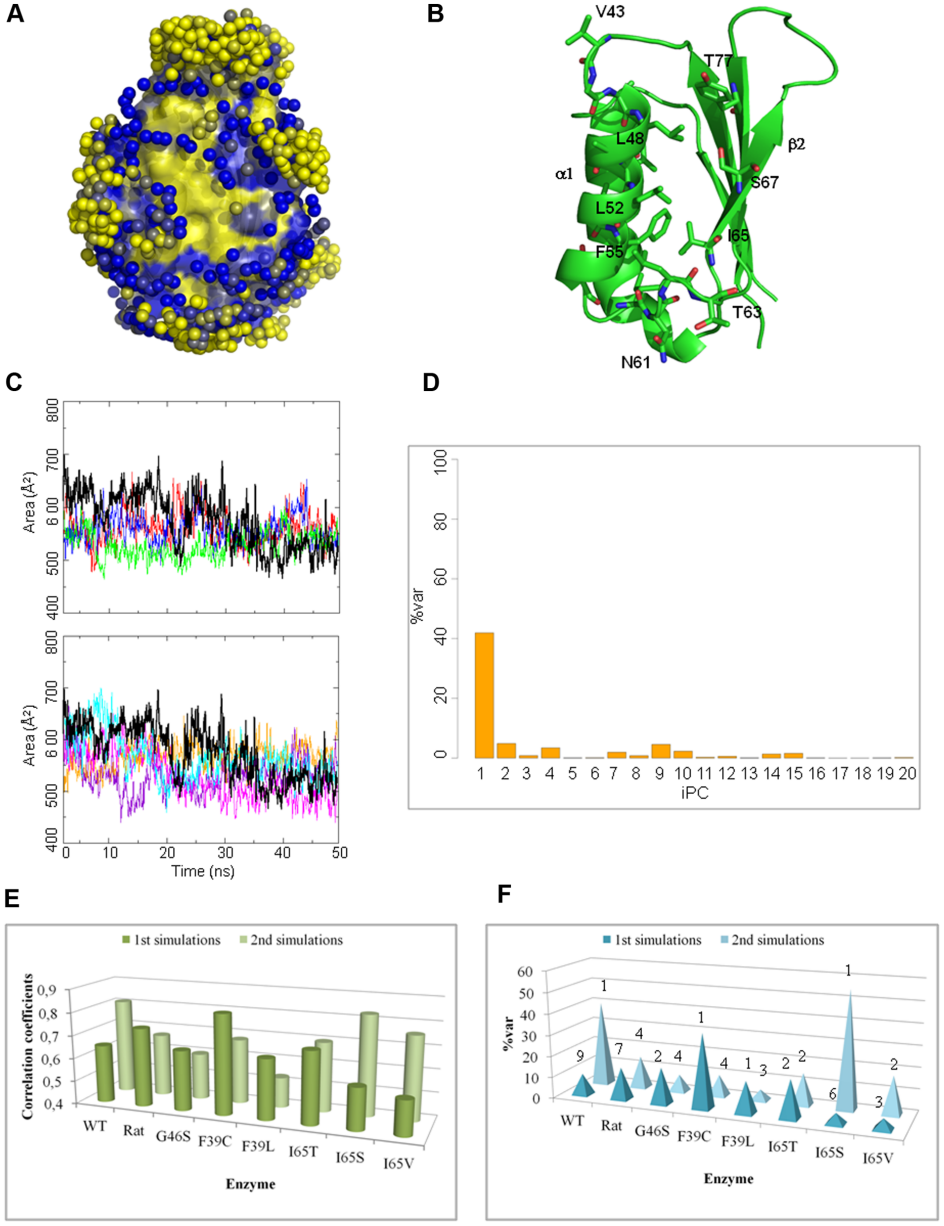
Analysis of the hydrophobic surface between helix α1 and the β2-strand. A) Water distribution in the wt-hPAH. The protein surface atoms are colored from yellow to blue according to the increasing hydration score *S_hyd_^atom^*. The local maxima of the water density map used for the calculation of the score (hydration sites) are represented as spheres, colored from yellow to blue according to the increasing density value. Hydrophobic atoms (yellow surface) have low hydration scores and are poor in hydration sites. B) The hydrophobic residues that belong to the hydrophobic surface between helix α1 and the strand β2 are shown as sticks in the wt-hPAH. C) Variation of the solvent accessible surface area (SASA) of the residues (backbone and side chains) at the hydrophobic surface between helix α1 and the β2-strand in the wt-hPAH (black) and in the mutants (G46S in orange, F39C in blue, F39L in green, I65T in violet, I65S in magenta, I65V in cyan). D) Relative contribution of the first 20 PCs in the maximally correlated motion to the variance of the hydrophobic surface SASA in wt-hPAH. E) Plot of the Pearson correlation coefficients between the trajectory projection on the MCM and the SASA of the hydrophobic surface (C), for all the systems and replicas. F) Plot of the maximum %var per PC for each system and replicas. The PC that has the largest relative contribution is indicated for each replica.

An extended hydrophobic surface between helix α1 and strand β2 was identified by analyzing the water density distribution around the solutes during MD simulations. The water density maps show the presence of a reduced number of hydration sites near this surface in all systems (Figure 6A). We calculated the variation of the solvent accessible surface area (SASA) of this region (residues Val45-Ala49, Val51, Leu52, Leu54, Phe55, Val60-Thr63, Ile65, Ser67, Tyr77 [Figure 6B]) during simulations. In the wt-hPAH a significant decrease of the SASA was observed, while in the simulations of the mutants the SASA values generally showed slight variations (Figure 6C).

To determine the correlation between the SASA variation of the identified hydrophobic surface and the protein collective motions, we carried out an FMA on all the simulations (see Materials and Methods). The collective motion that was maximally correlated with the SASA (MCM) was expanded in the space of the first n PCs that account for 90% of the overall fluctuation (essential space). In the case of the wild-type enzyme, an overall MCM-SASA correlation coefficient of 0.82 was observed for the second replica (Figure 6E), with PC1 accounting for more than 40% of the SASA variance (%var in Figure 6D). This indicates that the reorganization of the hydrophobic patch during the simulation was mainly related with the motion described by PC1 (Figure 2C), and in particular with the restructuring of the L2 loop, which hosts some of the residues in the patch. The mutants showed correlation values ranging from 0.53 to 0.83, with only F39C and I65S having correlations as high as the human wild-type enzyme (Figure 6E). The MCM expansion of these two mutants was dominated by PC1 (Figure 6F), analogous to the human wild-type enzyme. Although the F39C mutant did not show elongation of the β2-strand, the PC1 of its trajectory describes a large movement of the L2 and L4 regions that approach each other, as in the wt-hPAh and in the I65S mutant. This motion seems to modify the hydrophobic surface. In the case of the rat wild-type enzyme and the other human mutants, no dominant contributions to the SASA variation were observed across the essential space (Figure 6F).

### Analysis of Core Interactions

**Figure 7:**
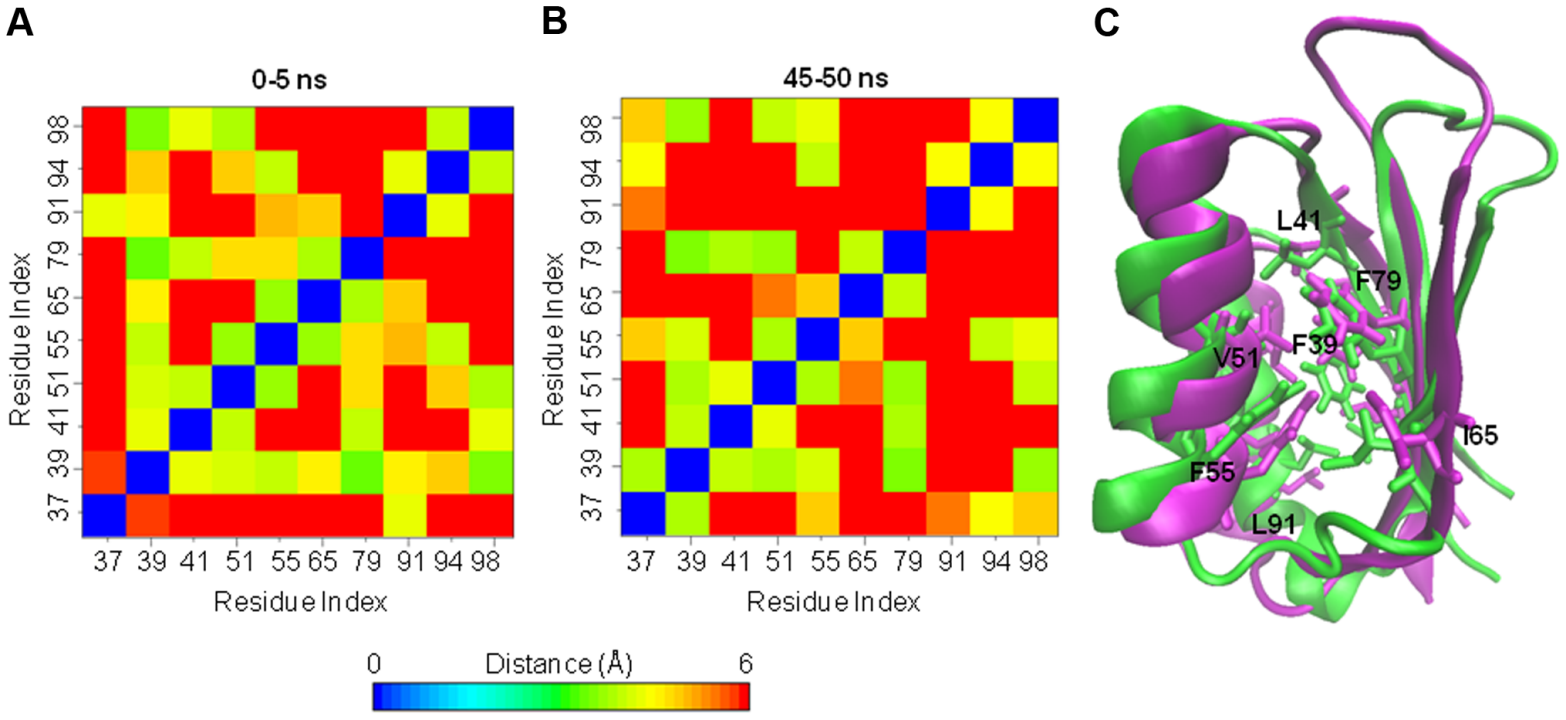
Average distances between selected residues of the hydrophobic core in the wt-hPAH. A) Distance matrices calculated on selected residues (Leu37, Phe39, Leu41, Val51, Phe55, Ile65, Phe79, Leu91, Ile94, Leu98) of the hydrophobic core in the wt-hPAH. Average distances calculated over the first (0–5 ns) and B) the last (45–50 ns) 5 ns of simulation are reported. Distances between pairs of residues are calculated as the minimum over all possible pairs of non-hydrogen atoms of the side chain. C) Superimposition of the structure at 0 (green) and 50 (magenta) ns of wt-hPAH with the hydrophobic core residues shown as sticks. Selected residues are labelled.

The hydrophobic contacts formed by selected residues in the hydrophobic core of wt-hPAH (Leu37, Phe39, Leu41, Val51, Phe55, Ile65, Phe79, Leu91, Ile94, Leu98) were analyzed by calculating distance matrices averaged over the first 5 ns and over the last 5 ns of the simulations (Figure 7). The side chains of these residues undergo significant rearrangement during simulation. In particular, the distances between residues Leu37 (β1) and Phe39 (β1) on one side, and Leu91 (α2), Ile94 (α2) and Leu98 (α2) on the other, change due to unwinding of the α2 N-terminal part. Moreover, residue Ile65 (β2) changes its distance from Phe39 (β1), Phe55 (α1) and Leu91 (α2) due to elongation of the β2 strand.

**Figure 8:**
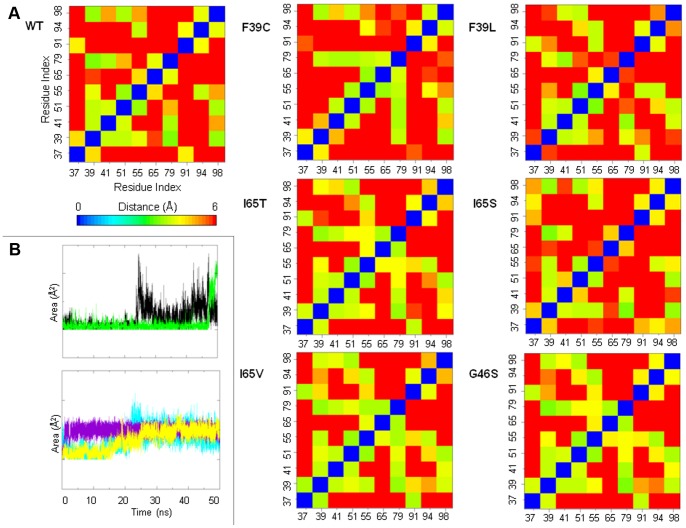
Analyses on the side-chains of the hydrophobic core residues in the wt-hPAH and in the mutants. A) Distances between pairs of selected hydrophobic core residues (Leu37, Phe39, Leu41, Val51, Phe55, Ile65, Phe79, Leu91, Ile94, Leu98) were calculated as the minimum over all possible pairs of non-hydrogen atoms of the side chain. Average values calculated over the 50-ns simulation are reported. B) Time evolution of the solvent accessible surface area (SASA) of Phe55 (black) and Val60 (green) in the F39C mutant (left, top). Time evolution of the SASA of the residue in position 65 in the I65S (yellow), I65T (violet) and I65V (cyan) mutants (left, bottom).

In the F39C and F39L mutants, the replacement of Phe39 with either a polar Cys or a smaller hydrophobic residue such as Leu, induced reorganization of the interactions in the core with respect to the wild-type enzyme. In particular, residue 39 lost its contacts with other core hydrophobic residues, namely Leu41, Val51 and Phe55 in F39C, and Leu37, Phe55 and Leu98 in F39L (Figure 8A). In the latter mutant, the distance between Ile65 and Phe79 was increased as a result of the L2 transition to a β-strand. The perturbation of the core is particularly evident at the end of the F39C simulation, when the two innermost residues (Phe55 and Val60) become exposed to the solvent in response to the movements of the L2 loop and of the α1 helix (Figure 8B).

In mutants of residue 65, the substitution with either a polar or a smaller hydrophobic residue may distort the hydrophobic packing in the RD core. In I65T and I65V, the side chains of some residues (Phe39, Val51, Phe55, Phe79, Ile94) of the hydrophobic core undergo a rearrangement during simulation and the distances between such residues differ with respect to the wild-type enzyme (Figure 8A). Unlike I65T and I65V, in the I65S mutant the contacts of residue 65 with the rest of the hydrophobic core are lost (Figure 8A). In all these mutants, the residue in position 65 becomes more exposed to solvent (Figure 8B) in response to elongation of β2.

In the G46S mutant, the Gly residue of the GAL ligand-binding motif of the ACT domain family is replaced by a polar residue. In this mutant some changes occur due to reorganization of the core side chains (Figure 8A), which adopt different orientations with respect to the wild-type enzyme during simulation. The average distance matrix of hydrophobic core residues for this mutant is similar to that of the I65T and I65V mutants (Figure 8A) thereby indicating that a similar reorganization of the core side chains is taking place.

**Figure 9:**
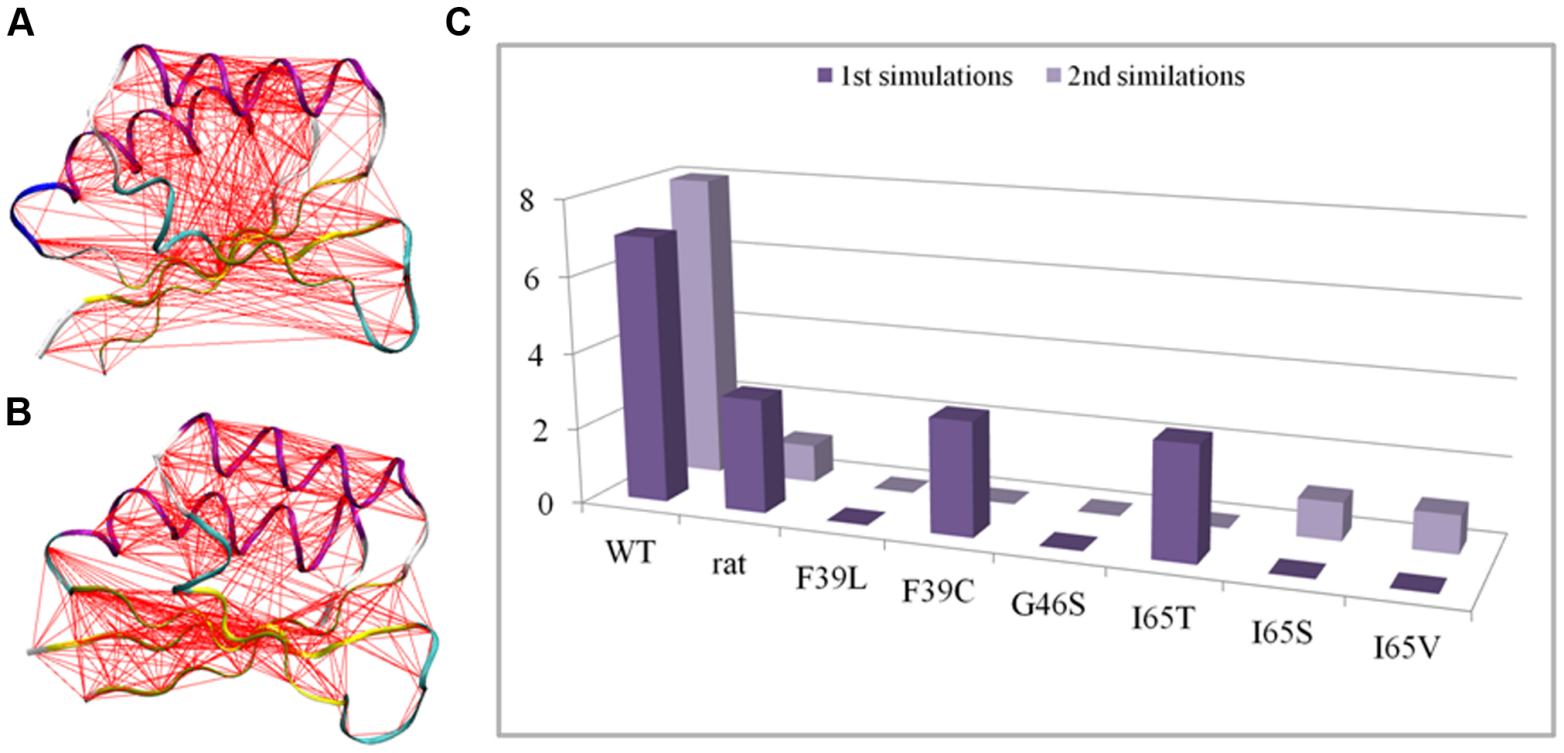
Correlation webs and number of links between helix and β-sheet residues. A and B) Correlation webs of wt-hPAH (A) and I65S (B) generated with Dynatraj. C^α^ atom pairs with a dynamic correlation higher than 0.3 are connected with a line. C) Total number of links connecting helix to β-strand residues in the correlation webs. Values from both replicas in each simulated system are reported.

Perturbation of the core introduced by the mutations is also evident from the comparison of the correlation webs of the different systems in which residues with dynamic correlations higher than 0.3 are connected by a line (Figures 9A, B). The wt-hPAH web (Figure 9A) shows a dense network of connections between the two helices on one side and the β-sheet on the other, which indicates that the two parts of the protein move concertedly in the main collective motions. All the mutant webs show a more disconnected pattern (see I65S in Figure 9B for an example), with a reduced number of links connecting helices with β-sheets (Figure 9C).

## Discussion

We carried out MD simulations on the isolated ACT regulatory domain of human and rat wild-type PAH and of six human disease-causing mutants. The analysis of the trajectories provided structural and dynamical insight into the isolated RD and into the effects of the mutations. We simulated only the structured regions of the RD (residues 33–111) because the N-terminal part is partially unstructured (residues 1–32). The structured regions of the RD coincide with the structural motif known as the “ACT domain” that consists of a four-stranded antiparallel β-sheet, two short α-helices and four connecting loops. Examination of the 50-ns-long MD trajectories revealed a specific dynamic behavior of the wild-type enzymes and the mutants. The simulated systems, while preserving their overall secondary structure, showed significant rearrangements of the loop-containing regions.

In 10 out of 16 simulations, the L2 region, which in the rat crystallographic structure (and in the generated models) has a turn/bend conformation, underwent a conformational change to a β-strand conformation that elongates the β2 strand. Elongation of β2 is associated with the formation of a typical H-bond pattern with the adjacent β3 that leads to the stabilization of the β-sheet. This suggests that the β-strand conformation of the L2 region is an accessible and intrinsically stable conformation of the RD. This finding is supported by the similarity with the known structure of the other ACT-domain-containing proteins and by the stability of the L2 β-strand conformation during 50-ns-long MD simulations of a model of the rat PAH, obtained using the ACT domain from 3PGDH with an elongated β2-strand as template. The conformational change of the L2 region leads to the stabilization of the β2-strand formed by residues 65–69. These residues constitute the conserved motif IESRP involved in the putative Phe binding at the isolated PAH RD. In addition, residues 60–63 in the L2 region show a high degree of evolutionary conservation [26]. A large portion of mutations leading to HPA cluster within or around the β2-strand [26], suggesting that these residues are relevant for the activity of the enzyme.

The principal motion in most of the studied systems involves a correlated conformational change in both the L2 and L4 regions. The nearing of L2 and L4, together with the unwinding of the N-terminal part of the α2 helix promotes the formation of H-bonds between L2 and L4. Collectively, all these movements result in a transition from an initial open to a final more closed structure of the domain, with the L2 and L4 regions, and the N-terminal part of β2 and the C-terminal part of β3 closer together.

Our findings are in agreement with the hypothesis formulated by Li and co-workers on the basis of hydrogen/deuterium exchange experiments conducted on PAH [54]. These authors concluded that upon Phe-binding (i) the interactions between the two peptides 39–59 and 82–91 are partly disrupted, leading to a more open RD, and (ii) the interaction between the RD and the catalytic domain is altered [54]. Interestingly, the 39–59 and 82–91 peptides partially and completely coincide with the dynamic L2 and L4 regions, respectively.

In addition, the analysis of our trajectories revealed a hydrophobic patch in the region between helix α1 and the β2 strand that corresponds to the ligand binding-site of the ACT protein family. This could be a site of interaction for the benzyl side chain of the Phe ligand. We found that the solvent accessibility of the hydrophobic surface is correlated to the global intrinsic motions of the protein and, particularly in the case of the wild-type enzyme, to the motions of the L2 and L4 regions, which suggests a relationship between Phe-binding and the overall dynamic behavior of the enzyme. The transition from an open to a closed form of the domain leads to a decrease of solvent exposure of the hydrophobic surface where the Phe ligand could bind to the domain. This suggests that the closed form of the enzyme, with a smaller hydrophobic surface, is unbound, while the open form is able to bind Phe. This is in agreement with the regulatory model in which PAH switches from an inactive to an active form upon Phe binding [20], [21].

The difference between the rat and human PAH RD dynamics might be related to the difference in the activity of the two enzymes. Pre-treatment of the rat enzyme with Phe is reported to produce a 10- to 30-fold increment of the activity versus an increase of only ∼3 to 6-fold in the human enzyme [21], [55]. This is due to the higher levels of activity of the not Phe-pretreated human enzyme, which probably undergoes different conformational changes with respect to the rat enzyme in the activation process.

**Figure 10:**
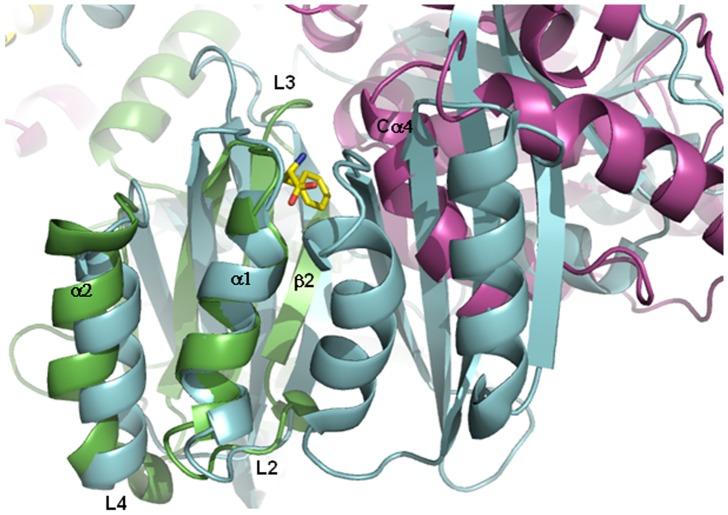
Superimposition of the Phe-bound prephenate dehydratase (PDT) and the tetrameric model of the hPAH. The Phe-bound PDT from *Chlorobium tepidum TLS* (PDB code 2QMX) is drawn in cyan. The RD of a subunit of hPAH is drawn in green, whereas the catalytic domain of the adjacent subunit is drawn in violet. The bound Phe ligand is shown as a yellow stick.

To compare in greater detail the binding site of the ACT domain-containing proteins and the corresponding region in hPAH, we generated an *in silico* model of a hPAH tetramer (see Materials and Methods) and subsequently superposed it to Phe-bound PDT (PDB code 2QMX). The tetrameric model provided information on the position of the RDs within the entire enzyme. Moreover, the superposition revealed a PDT Phe-binding site located at the interface between the RD and the catalytic domain of the adjacent subunit in PAH, in correspondence to the hydrophobic surface on the RD which harbors the putative Phe binding site. In particular, an α-helix (Cα4, according to the secondary structure notation by Flatmark et al. [56]) from the catalytic domain in PAH is located in a similar position to the Phe-binding site region of the second ACT domain in PDT (Figure 10). Thus, some residues of this α-helix could participate in Phe-binding in PAH, probably through conformational changes in the catalytic domain and reorientation of the two interacting domains upon Phe-binding.

Taken together, our simulations provide insight into the functional relevance of the intrinsic dynamics of the isolated PAH RD, showing the possible motions responsible for the modulation of the putative Phe-binding site involved in PAH allosteric regulation. A future study of the allosteric mechanism would require enhanced sampling MD techniques [57] to accelerate the exploration of the conformational space either of a full-length subunit, or of a dimer relevant for PAH allosteric regulation.

To obtain insight into the structural role of HPA-disease-causing mutations in the RD hydrophobic core, we examined the F39C, F39L, I65T, I65S and I65V mutants. Residues 39 and 65 are the most frequently mutated positions and their resulting phenotypes can range from mild to severe PKU (http://www.pahdb.mcgill.ca/). In addition, we examined the G46S mutant, which is associated with a severe form of PKU [14]. Residue Gly46, located between the β1-strand and the α1 helix, is a good candidate for Phe binding since it lies in the ligand-binding site in other ACT domain-containing proteins [17]. The mutants show residual activity, namely, between 60% and 67% of the wild-type enzyme, and display various kinetic defects, such as reduced Phe activation and reduced cooperativity of substrate binding (Table S2) [14], [28], [58], [59].

In all mutants, the extent of correlation of motion between the helix and β-sheet residues was smaller than in the human wild-type enzyme. In mutants G46S, F39L, I65T and I65V, also the correlation between the variation of the hydrophobic surface and the movements of the L2 and L4 regions was smaller than the wild-type enzyme. Taken together, these data suggest that changes in the interactions within the hydrophobic core of the RD may significantly affect its stability and hence its regulatory role. Indeed, changes in RD stability could affect the cross-talks with the other domains and interfere with the conformational changes that are involved in cooperativity.

In conclusion, we hypothesize a mechanism by which alterations in the hydrophobic interactions of the RD may hamper the functionality of the whole enzyme. The RD mutations can influence the correct binding of Phe to the domain thereby resulting in poor Phe activation that ultimately reduces enzyme activity. This is in line with experimental data on the mutants that have been characterized *in vitro* showing reduced Phe activation and reduced activity (Table S2) [26], [28], [58], [60].

## Supporting Information

Figure S1 **Root Mean Square Fluctuation (RMSF) of the C^α^ atoms during the simulation.** Color code: wt-hPAH in black, wt-rPAH in red, G46S in orange, F39C in blue, F39L in green, I65T in violet, I65S in magenta, I65V in cyan.
(TIFF)

Figure S2 **Secondary structure analysis.** Time-evolution of the DSSP secondary structure for the human (WT) and rat (rat X-ray) wild-type enzymes, the human mutants, and the *in silico* model of the rat enzyme using as template the ACT domain of 3PGDH (rat model).
(TIFF)

Figure S3 **Time-evolution of H-bond number between the β2 strand (65–69 residues) and the β3 strand (76–81 residues) in the wt-hPAH.** Hydrogen bonds are determined between the backbone atoms using a cutoff of 30 degrees on the Acceptor – Donor – Hydrogen angle and of 3.5 Å on the Acceptor - Donor distance.
(TIFF)

Table S1 **Root mean square deviations (RMSD) with standard deviations (SD) of all studied PAH forms for both replicas.**
(DOCX)

Table S2 **Kinetic parameters of the hPAH studied forms.**
(DOCX)
