# Supplementary figures and images for: Structural Features of the Regulatory ACT Domain of Phenylalanine Hydroxylase
Source: PLoS One. 2013 Nov 14;8(11):e79482. doi: 10.1371/journal.pone.0079482 (PMC3828330; doi:10.1371/journal.pone.0079482)

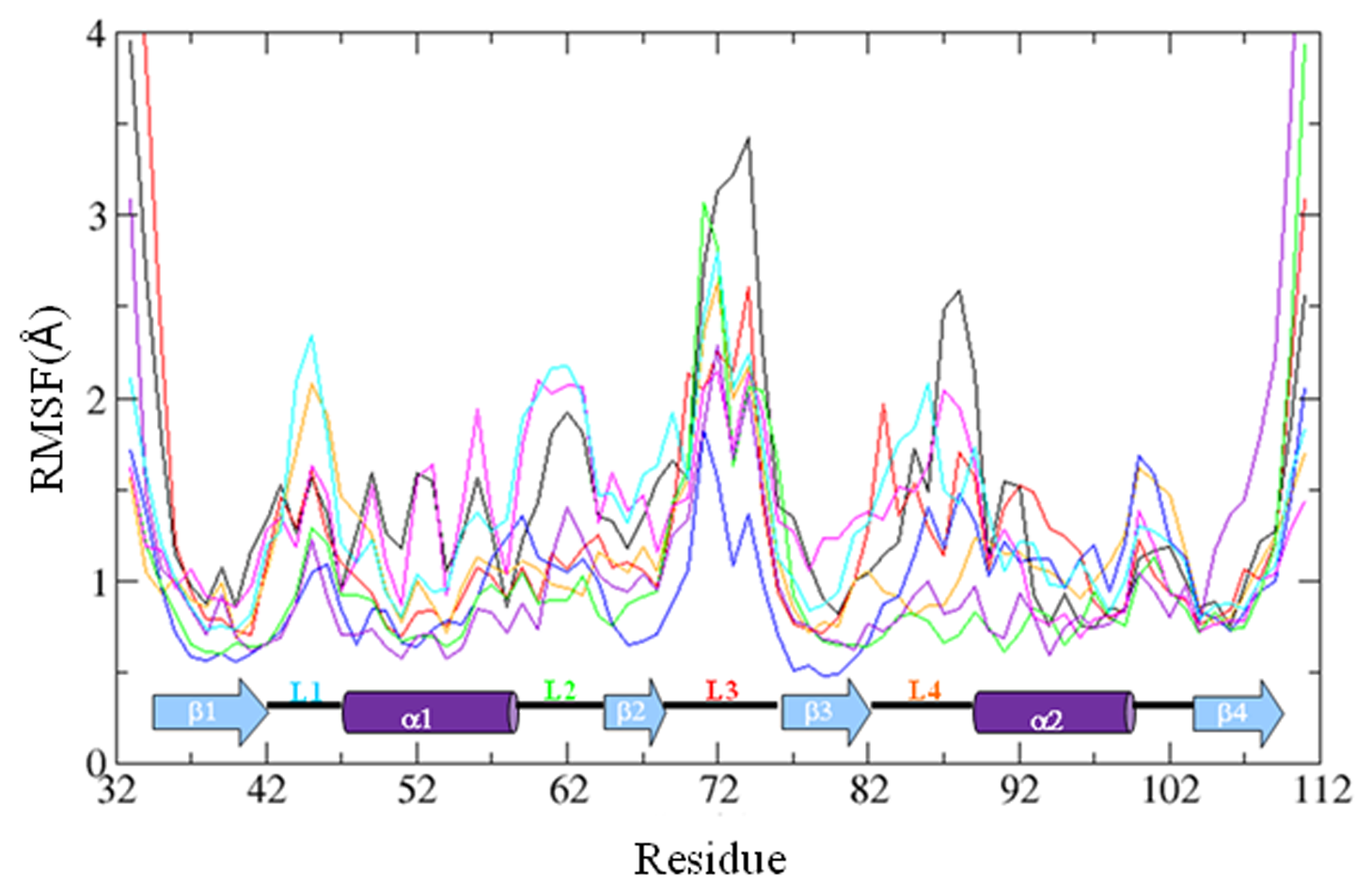

Supplement: Figure S1 — Root Mean Square Fluctuation (RMSF) of the Cα atoms during the simulation. Color code: wt-hPAH in black, wt-rPAH in red, G46S in orange, F39C in blue, F39L in green, I65T in violet, I65S in magenta, I65V in cyan. (TIFF) [file pone.0079482.s001.tiff]

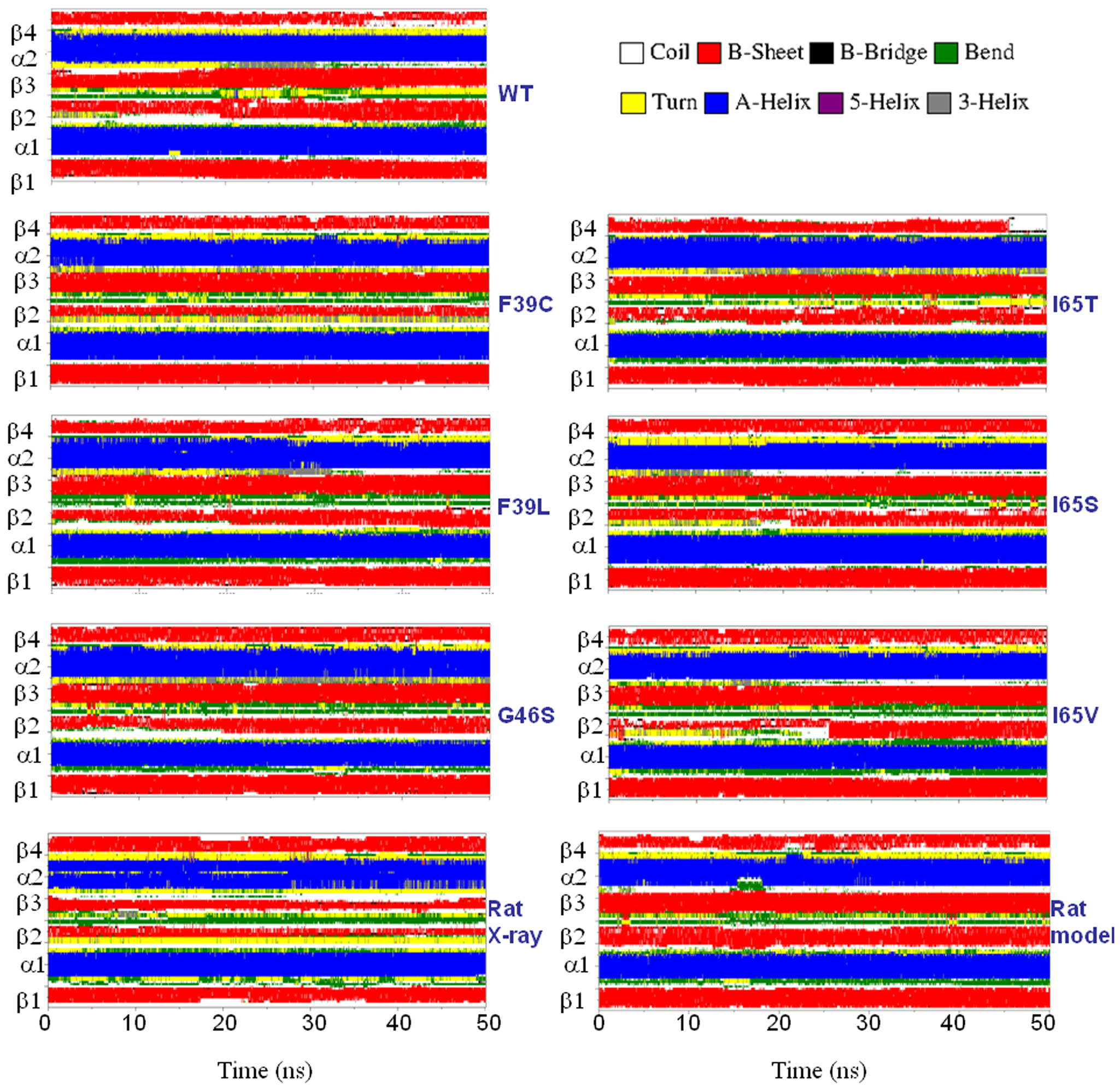

Supplement: Figure S2 — Secondary structure analysis. Time-evolution of the DSSP secondary structure for the human (WT) and rat (rat X-ray) wild-type enzymes, the human mutants, and the in silico model of the rat enzyme using as template the ACT domain of 3PGDH (rat model). (TIFF) [file pone.0079482.s002.tiff]

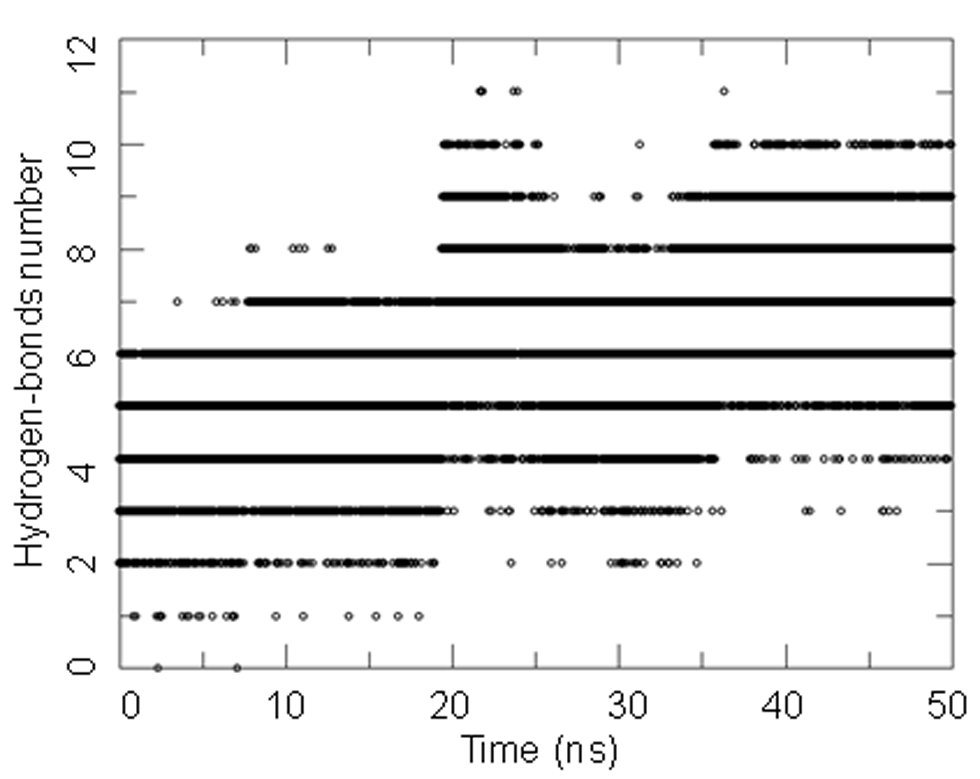

Supplement: Figure S3 — Time-evolution of H-bond number between the β2 strand (65–69 residues) and the β3 strand (76–81 residues) in the wt-hPAH. Hydrogen bonds are determined between the backbone atoms using a cutoff of 30 degrees on the Acceptor – Donor – Hydrogen angle and of 3.5 Å on the Acceptor - Donor distance. (TIFF) [file pone.0079482.s003.tiff]
