# Supplementary material for: Structural Features of the Regulatory ACT Domain of Phenylalanine Hydroxylase
Source: PLoS One. 2013 Nov 14;8(11):e79482. doi: 10.1371/journal.pone.0079482 (PMC3828330; doi:10.1371/journal.pone.0079482)
Supplement: Table S1 — Root mean square deviations (RMSD) with standard deviations (SD) of all studied PAH forms for both replicas. (DOCX) [file pone.0079482.s004.docx]

Table S1: Root mean square deviations (RMSD)

with standard deviations (SD) of all studied PAH

forms for both replicas.

| **PAH form** | **RMSD ± SD^1^ (Å)** | |
| --- | --- | --- |
|  | **1^st^ simulation** | **2^nd^ simulation** |
| Wt-hPAH | 2.81 ± 0.13 | 2.81 ± 0.16 |
| Wt-rPAH | 2.83 ± 0.14 | 2.32 ± 0.13 |
| F39C | 2.60 ± 0.25 | 1.73 ± 0.15 |
| F39L | 2.86 ± 0.14 | 3.23 ± 0.16 |
| G46S | 2.70 ± 0.20 | 2.96 ± 0.17 |
| I65S | 2.23 ± 0.14 | 2.73 ± 0.12 |
| I65T | 2.56 ± 0.10 | 2.32 ± 0.13 |
| I65V | 2.57 ± 0.13 | 2.30 ± 0.13 |

^1^RMSD values are calculated with respect to the

starting structures. The reported values are the

average calculated over the last 5ns of simulation.
