# Supplementary material for: Structural Features of the Regulatory ACT Domain of Phenylalanine Hydroxylase
Source: PLoS One. 2013 Nov 14;8(11):e79482. doi: 10.1371/journal.pone.0079482 (PMC3828330; doi:10.1371/journal.pone.0079482)
Supplement: Table S2 — Kinetic parameters of the hPAH studied forms. (DOCX) [file pone.0079482.s005.docx]

Table S2: Kinetic parameters of the hPAH studied forms.

| **PAH form** | **Frequency (%)^1^** | **Phenotype** | **Activity (as % of WT)** | **Activation fold^2^** | **Hill coefficient^2^** |
| --- | --- | --- | --- | --- | --- |
| WT |  |  | 100 | 2.6 [56] | 2.0 [56] |
| F39L  F39C | 1.31 | Mild to classical [28, 56] | 67 ± 8 [28] | 1.2 [56] | 1.4 [56] |
| G46S | 0.50 | Mild to severe [14, 57] | 62 ± 13 [28] | 1.1 [57] | 1.2 [57] |
| I65T  I65S  I65V | 4.06 | Non-PKU HPA to classical [28, 56] | 60 ± 25 [28] | 1.2 [56] | 1.0 [56] |

**^1^**The frequency is taken from the PAH Mutation Analysis Consortium database (<http://www.pahdb.mcgill.ca/>). The frequency of each mutation has been provided through statistics analysis on the entire set of mutations reported in the database; i.e. the mutation I65T is the 3^rd^ more frequent and is the most frequent one among those falling in the RD.

**^2^**The response to Phe binding is reported both as activation fold by preincubation with the substrate and as degree of positive binding cooperativity (Hill coefficient).
